# Zebularine Promotes Hepatic Differentiation of Rabbit Bone Marrow Mesenchymal Stem Cells by Interfering with p38 MAPK Signaling

**DOI:** 10.1155/2018/9612512

**Published:** 2018-10-10

**Authors:** Yong-Heng Luo, Juan Chen, En-Hua Xiao, Qiu-Yun Li, Yong-Mei Luo

**Affiliations:** ^1^Department of Radiology, Second Xiangya Hospital of Central South University, Changsha, Hunan 410011, China; ^2^Department of safety & environmental protection, Shenzhen Zhongjin Lingnan Nonfemet Company Ltd, Shenzhen, Guangdong 518040, China

## Abstract

Demethylating agent zebularine is reported to be capable of inducing differentiation of stem cells by activation of methylated genes, though its function in hepatocyte differentiation is unclear. p38 signal pathway is involved in differentiation of hepatocytes and regulating of DNA methyltransferases 1 (DNMT1) expression. However, little is known about the impact of zebularine on bone marrow mesenchymal stem cells (BMMSCs) and p38 signaling during hepatic differentiation. The present study investigated the effects of zebularine on hepatic differentiation of rabbit BMMSCs, as well as the role of p38 on DNMT1 and hepatic differentiation, with the aim of developing a novel strategy for improving derivation of hepatocytes. BMMSCs were treated with zebularine at concentrations of 10, 20, 50, and 100 *μ*M in the presence of hepatocyte growth factor; changes in the levels of hepatic-specific alpha-fetoprotein and albumin were detected and determined by RT-PCR, WB, and immunofluorescence staining. Expression of DNMT1 and phosphorylated p38 as well as urea production and ICG metabolism was also analyzed. Zebularine at concentrations of 10, 20, and 50 *μ*M could not affect cell viability after 48 h. Zebularine treatment leads to an inhibition of DNMT activity and increase of hepatic-specific proteins alpha-fetoprotein and albumin in BMMSCs in vitro; zebularine addition also induced expression of urea production of and ICG metabolism. p38 signal was activated in BMMSCs simulated with HGF; inhibition of p38 facilitated the synthesis of DNMT1 and albumin in cells. Zebularine restrained DNMT1 and phosphorylated p38 which were induced by HGF. Therefore, this study demonstrated that treatment with zebularine exhibited terminal hepatic differentiation of BMMSCs in vitro in association with hepatocyte growth factor; p38 pathway at least partially participates in zebularine-induced hepatic differentiation of rabbit BMMSCs.

## 1. Introduction

Liver regeneration is critical for graft survival and competent organ function. Bone marrow mesenchymal stem cells (BMMSCs) have immense potential in the field of regenerative medicine for its multilineage potential. BMMSCs, therefore, are increasingly being considered in cell-based therapeutic strategies for liver regeneration. However, their low hepatic differentiation efficiency in vitro is a significant obstacle to hepatocyte transplantation. Current evidence suggests that epigenetic programming plays an important role in stem cell biology by maintaining pluripotency and promoting stem cell differentiation into more mature cell progenies [1, 2]. A key epigenetic reprogramming mechanism is DNA methylation. DNA methyltransferase inhibitors (DNMTi) initiate differentiation by decreasing the global methylation status of the cells, and therefore, genes are more prone to transcription. Recently, it was shown that DNMTi, such as 5-azacytidine and zebularine, are capable of inducing the differentiation of stem cells [3–6]. Though previous studies have demonstrated that treatment of BMMSCs isolated from different sources with 5-azacytidine resulted in the expression of hepatic-specific proteins in these cells in vitro [6, 7], its use can be limited by its instabilities and toxicities. Zebularine (1-(*β*-d-ribofuranosyl)-1, 2-dihydropyrimidin-2-one) is a second-generation hydrophilic inhibitor of DNA methylation [8]. In contrast to 5-azacytidine and other DNMTi, zebularine is quite stable [9] and has low toxicity [10], making it an attractive candidate for liver regeneration. However, to date, no data have been published regarding the course of action of zebularine in liver regeneration. It would be worthwhile to investigate whether treatment of BMMSCs with zebularine can direct them toward specific hepatic lineage.

Hepatocyte growth factor (HGF) played a critical role in the development and regeneration of the liver and induced expression of the hepatic phenotype in mesenchymal stem cells in vitro [11, 12]. Mitogen-activated protein kinases (MAPKs) play important roles in the cellular response to growth factors, cytokines and chemicals, or environmental stress. p38 signal pathway is one of the major MAPK pathways. In addition to inflammation and stress responses, p38 is involved in the control of cell cycle and proliferation [13]. Previous studies have indicated the reciprocal regulation of HGF and activated p38 in cell proliferation [14–16]. Transforming growth factor-*β* (TGF-*β*) downregulated activation of HGF precursors and influenced hepatocyte plasticity via p38 signal pathway [15, 17, 18]. Besides that, HGF phosphorylated p38, but p-p38 suppressed the activation of DNA synthesis by HGF, while the p38 inhibitor SB203580 increased HGF-induced DNA synthesis [19]. Taken together, the data presented here highlight the important role that p38 signal pathway plays in the differentiation and proliferation of hepatocytes. However, little is known about the impact of zebularine on HGF-induced hepatic differentiation and p38 activation.

In this study, we investigated the effects of zebularine on DNA methyltransferases 1 (DNMT1) and hepatic differentiation of BMMSCs. Activation of p38 and its role on DNMT1 and hepatic differentiation were also analyzed. The objective is to come up with a novel strategy that would be highly potent in terms of differentiating the BMMSCs into hepatocytes while being least toxic to biological systems.

## 2. Materials and Methods

### 2.1. Reagents

We purchased sheep anti-rabbit antibodies of CD29, CD34, CD44, CD45, and fluorescein isothiocyanate- (FITC-) labeled IgG secondary antibody from eBioscience (San Diego, CA). Sheep polyclonal anti-human antibodies to alpha-fetoprotein (AFP), albumin (ALB) and *β*-actin, and FITC-labeled IgG secondary antibody were obtained from Sigma (St. Louis, MO). Antibodies to DNMT1 and phosphorylated p38 were obtained from Cell Signaling Technology (Beverly, MA). HGF was purchased from Proteintech (Rocky Hill, NJ). Zebularine was purchased from Berry and Associates (Dexter, MI).

The p38 inhibitor SB203580 was obtained from Merck (Germany). A stock solution of SB203580 was prepared in dimethyl sulfoxide (DMSO). SB203580 was diluted in Dulbecco modified Eagle medium (DMEM; Gibco, Rockville, MD) and was added to cell culture 1 h before stimulation with HGF and kept further during the exposure to this compound. A stock solution of zebularine for cellular assays was prepared in DMSO and then diluted in the optimal medium to the final concentrations. Indocyanine green (ICG) was obtained from Aladdin (Shanghai, China).

### 2.2. Cell Culture

The biological study was approved by the Medicine Human Ethics Committee of the Second Xiangya Hospital of the Central South University. Rabbit bone marrow was obtained from three New Zealand rabbits (male, two-month-old, less than 1 kg in weight) obtained from the Laboratory Animal Unit of the Second Xiangya Hospital of the Central South University. BMMSCs were harvested from expansion culture of cell suspension and were propagated in DMEM supplemented with 10% fetal bovine serum (FBS; Gibco, Rockville, MD), 100 U/ml penicillin, and 100 mg/ml streptomycin. The third to fifth generation of cells were collected for study. BMMSCs were identified by the flow cytometric analysis of CD29, CD34, CD44, and CD45. The experiments were performed at least three times in cell strains from the three rabbits and obtained similar results.

Primary rabbit hepatocytes were isolated from anesthetised rabbits by in situ perfusion of the liver with 0.05% collagenase as previously described [20]. Cell suspensions were seeded in collagen-coated (100 mg) petri dishes at a concentration of 9 × 10^6^ viable cell in a 1 : 1 (v/v) Ham's F12 and William's E medium. Plates were thereby incubated at 37°C under 5% CO_2_ and 95% air. The culture medium was replenished every other day.

### 2.3. Flow Cytometry

Approximately 1 × 10^6^ cells of the third generation cells were harvested and resuspended in 1 ml phosphate-buffered saline (PBS). Cells were incubated with primary antibodies of CD29 (1 : 100), CD34 (1 : 100), CD44 (1 : 100), CD45 (1 : 100), and ALB (1 : 200) for 1 h at 4°C. Subsequently, the cells were washed with PBS for 3 times and incubated with FITC-conjugated secondary sheep antibody (1 : 200) for 40 min at 4°C.

### 2.4. Treatment of BMMSCs with Zebularine

Cytotoxicity of zebularine was evaluated by trypan blue exclusion test prior to the experiment. Six concentrations (0, 10, 20, 50, 100, and 200 *μ*M) of zebularine were used for assessment. Zebularine was supplemented to cells to final concentrations and incubated in CO_2_ incubator at 37°C. Cell viability was measured after treatment for 48 h. A hemocytometer was used to count the unstained (viable) and the stained (dead) cells. The percentage of cell viability was calculated according to the formula cell viability (%) = (the number of the viable cells/total cells) × 100%.

HGF (60 *μ*g/l) was utilized as the hepatocyte-inducing protocol. BMMSCs were seeded in cell culture flasks and grown to subconfluence. The cells were incubated with zebularine (0, 10, 20, 50, and 100 *μ*M) in the presence of HGF for 24 h at 37°C in a humidified atmosphere containing 5% CO_2_. After incubation, the medium was aspirated, and the cells were then washed twice and cultured in medium with HGF. The noninduced BMMSCs and primary rabbit hepatocytes were used as negative or positive controls. The experiments were terminated after 21 days.

### 2.5. Quantitative Real-Time Polymerase Chain Reaction (RT-PCR)

Cells in each group were collected on days 7, 14, and 21. The total RNA was harvested using Trizol according to instructions of the manufacturer (Invitrogen, Carlsbad, CA). After RNA gel electrophoresis, the qualified RNA was subjected to reverse transcription using the PrimeScript RT reagent kit (Takara, Shiga, Japan). Polymerase chain reaction conditions were initial denaturation at 95°C for 10 min, followed by 40 cycles at 95°C for 5 s and 60°C for 30 s. Amplification of the housekeeping gene *β*-actin mRNA was carried out with forward (CATCCTGCGTCTGGACCTGG) and reverse (TAATGTCACGCACGATTTCC) primers. The forward and reverse primer sequences were CCCTCATCCTCCTGCTACATT and CGGAACAAACTGGGTAAAGGT for AFP, AAGACGTGTGTTGCCGATGA and GCCTTTCAAATGGCAGG for ALB. The comparative threshold cycle (Ct) method was used to determine the relative ratio of expression for each gene.

### 2.6. Western Blot

Cell proteins were extracted using lysis buffer (20 mM Tris, 150 mM NaCl, 1 mM EDTA, 1% Triton X-100) supplemented with phosphatase inhibitors (1 mM sodium vanadate, 50 mM NaF) and protease inhibitors (0.1% phenylmethylsulfonyl fluoride; Complete protease inhibitor, Roche). After being boiled in Laemmli sample buffer, 10 mg of protein extracts was subjected to SDS-PAGE gel. Next, proteins were transferred to a polyvinylidene difluoride membrane by a Bio-Rad gel-blotting apparatus (Bio-Rad, Hercules, CA, USA). The blots were blocked with 5% nonfat dry milk at room temperature for 1 h and then incubated overnight with the desired primary antibodies at 4°C. After being washed thrice for 10 min each with TBST, peroxidase was detected by chemiluminescence and visualized by exposure to X-ray films (Kodak, USA). Quantification of Western blot products was performed by a laser densitometry (Bio-Rad, USA) and presented as a ratio between optical density of target protein band and *β*-actin band.

### 2.7. Immunofluorescence Staining

After being rinsed in PBS, cells cultured on coverslips in each group were fixed in 40 g/l paraformaldehyde, permeabilized by triton X-100, and blocked in phosphate-buffered saline buffer supplemented with 5% bovine serum albumin. Cells were labeled with primary sheep anti-rabbit antibodies against ALB (1 : 100) for 1 h at 4°C. After washing, cells were incubated with FITC-conjugated secondary antibodies for ALB. DAPI was used as a nuclear counterstain. Cells were visualized using a fluorescence microscope (Axio Vert 200, Zeiss, Germany).

### 2.8. ICG Uptake

ICG was dissolved in 0.01 M PBS to produce a 5 mg/ml stock solution and was diluted in optimal medium to a final concentration of 1 mg/ml. The seeded scaffolds were immersed in the ICG solution and incubated for 30 min at 37°C. After being washed using PBS, the cells were finally examined and imaged microscopically.

### 2.9. Urea Production

The differentiated BMMSCs were incubated with 1 ml medium containing 5 mM NH4Cl (Sigma) for 24 h at 37°C on days 0, 7, 14, and 21. After incubation, the supernatants were collected and the urea concentrations were analyzed using a colorimetric assay kit (GBD Corporation, San Diego, CA). The content of urea was presented in terms of pg protein/cell/day.

### 2.10. Statistical Analysis

All data are presented as means ± SD. Statistical analyses were performed with SPSS 19.0 (Chicago, IL, USA). Comparisons between results from multiple treatment groups were performed using one-way analysis of variance (ANOVA), and LSD test was performed as a post hoc test to show individual differences. Statistical significance was defined as *p* < 0.05.

## 3. Results

### 3.1. BMMSC Phenotyping

**Figure 1:**
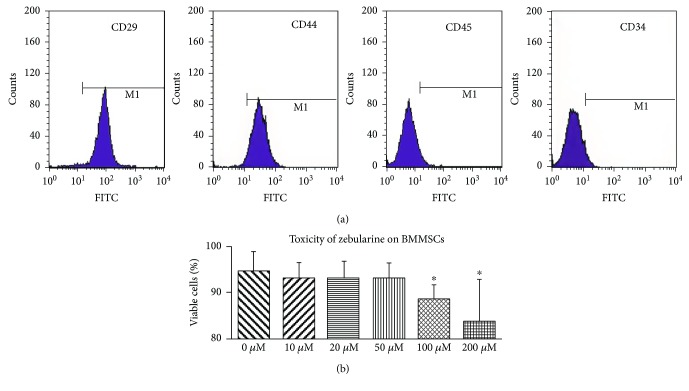
Positive expression rates of BMMSC surface marker antigens CD29, CD44, CD45, and CD34 and toxicity of zebularine on BMMSCs. Asterisk indicates *p* < 0.05 compared with the 0 *μ*M group.

The BMMSCs are characterized by expression of CD29 but negative for CD45. To address the cell phenotyping, flow cytometry analysis of CD29 and CD45 was performed. The cells showed high expression of CD29 (93.99% ± 3.21%) and CD44 (90.57% ± 3.03%) while there was no obvious expression of CD34 (3.08% ± 1.66%) and CD45 (3.81% ± 1.95%, Figure 1(a)).

### 3.2. Toxicity of Zebularine on BMMSCs

Cell viability in 200 *μ*M group is lower than that in 0, 10, 20, 50, and 100 *μ*M groups (all *p* < 0.05, Figure 1(b)), which means concentration of 200 *μ*M is toxic to cells. Cell viability in 100 *μ*M group is lower than that in 0 *μ*M group (*p* < 0.05); however, the difference is not significant when compared with control, 10, 20, and 50 *μ*M groups (all *p* > 0.05). There is no significant difference in the cell viability among 0, 10, 20, and 50 *μ*M groups (all *p* > 0.05) and for that reason were not considered as cytotoxic. Therefore, zebularine with concentrations of 0, 10, 20, 50, and 100 *μ*M was used in the subsequent studies.

### 3.3. Zebularine Induced the Differentiation of BMMSCs into Hepatocytes

**Figure 2:**
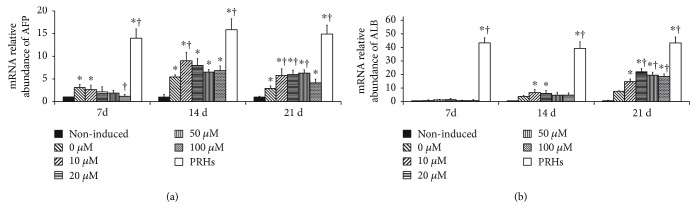
Zebularine promoted the mRNA expression of AFP and ALB. Noninduced BMMSCs and primary rabbit hepatocytes (PRHs) were used as negative or positive controls. (a) On day 7, noninduced BMMSCs expressed little AFP; increased AFP mRNA expression was observed in 0 and 10 *μ*M groups. On day 14, zebularine exhibited significant effect on AFP expression, with the highest expression level in 10 and 20 *μ*M groups. (b) Noninduced BMMSCs expressed little ALB. The mRNA levels of ALB started to increase at 14 days after pretreatment of zebularine at concentrations of 10 and 20 *μ*M. Exposure to zebularine exhibited significant effect on ALB expression after 21 days, with the highest expression level in the 20 *μ*M group. Asterisk indicates *p* < 0.05 compared with the control group; dagger indicates *p* < 0.05 compared with the 0 *μ*M (HGF) group.

AFP, a characteristic marker of hepatocyte during embryonic development and fetal stages, was recruited when hepatic transdifferentiation was studied. Noninduced BMMSCs expressed little AFP mRNA. Treatment with HGF in the absence of zebularine (0 *μ*M) increased the mRNA expression of AFP and reached a peak on day 14 (*p* < 0.05, Figure 2(a)). Pretreatment of zebularine at concentrations of 10 and 20 *μ*M also improved AFP expression on day 7 (*p* < 0.05, respectively), but there is no significant difference between the two concentrations (*p* < 0.05). On day 14, zebularine exhibited significant effect on AFP expression, with the highest expression level in 10 and 20 *μ*M groups, but there is no significant difference between 10 and 20 *μ*M groups (*p* > 0.05). Zebularine did not change the peak time. On day 21, mRNA expression of AFP decreased in all induced groups but still higher than that of noninduced group (*p* < 0.05, respectively), which indicated that induction of AFP persists long after the drug removal.

Differentiation of BMMSCs was also traced up by determining expression for ALB, a typical marker for mature hepatocytes. The mRNA expression of ALB increased on day 14 and reached a peak on day 21 in the 0 *μ*M group (*p* < 0.05, Figure 2(b)). Zebularine induced upregulation of ALB mRNA in a dose-dependent manner. Pretreatment with different doses of zebularine did not improve ALB expression on day 7 (*p* > 0.05, respectively). The mRNA levels of ALB started to increase at 14 days after stimulation of zebularine at concentrations of 10 and 20 *μ*M (*p* < 0.05, respectively). Exposure to zebularine exhibited significant effect on ALB expression after 21 days. With a decreased AFP expression and an increased ALB expression, BMMSCs were inclined to differentiate into mature hepatocyte-like cells. There were higher levels of AFP in 20, 50, and 100 *μ*M groups compared with that in the 0 *μ*M (HGF only) group (all *p* < 0.05), with the highest level in the 20 *μ*M group. Therefore, zebularine at a concentration of 20 *μ*M was used in subsequent experiments.

**Figure 3:**
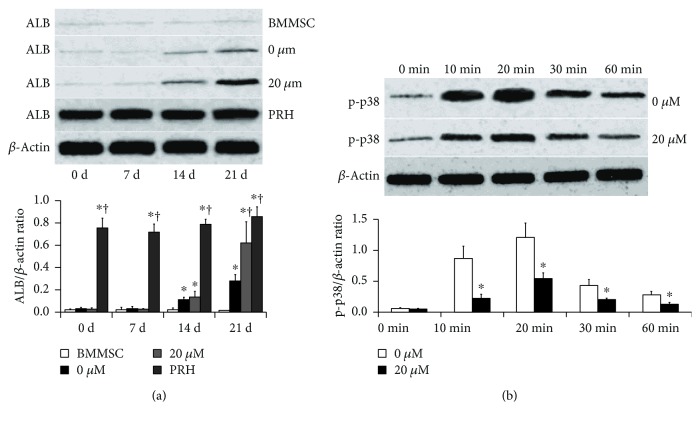
Zebularine promoted protein expression of ALB in BMMSCs and attenuated activation of the p38 signal pathway induced by HGF. (a) The protein expression of ALB increased on day 14 and reached a peak on day 21 in the 0 *μ*M (HGF only) group. Zebularine at 20 *μ*M clearly reinforced the HGF-induced upregulation of ALB protein expression. However, the peak of expression of ALB in both 0 and 20 *μ*M groups was lower than that in primary rabbit hepatocytes. (b) HGF (0 *μ*M group) increased the expression of phosphorylated p38 in cultured BMMSCs. However, addition of zebularine (20 *μ*M group) significantly suppressed p38 pathway activation in the cells stimulated with HGF. Asterisk indicates *p* < 0.05 compared with min 0; dagger indicates *p* < 0.05 compared with HGF (0 *μ*M group).

To further quantify the effects of zebularine on ALB expression, Western blot assays were performed. Noninduced BMMSCs expressed little ALB. The protein expression of ALB increased on day 14 and reached a peak on day 21 in the 0 *μ*M (HGF only) group (*p* < 0.05, Figure 3(a)). Zebularine at 20 *μ*M clearly reinforced the HGF-induced upregulation of ALB protein expression (*p* < 0.05). Furthermore, the peak of expression of ALB in the 20 *μ*M group (zebularine + HGF) was higher than that in the 0 *μ*M (HGF only) group (*p* < 0.05) on day 21, which followed the same trend with mRNA expression. However, the peak of expression of ALB in both 0 and 20 *μ*M groups was lower than that in primary rabbit hepatocytes (both *p* < 0.05).

### 3.4. Zebularine Inhibited Activation of the p38 Pathway in BMMSCs

To address the involvement of p38 in hepatic differentiation, phospho-specific antibody for the activated form of p38 was used in Western blot analyses of cell lysates. BMMSCs were stimulated with zebularine and/or HGF; the p-p38 level was measured at various time points from 0 to 60 min. HGF increased the expression of phosphorylated p38 in cultured BMMSCs. However, addition of zebularine significantly suppressed p38 pathway activation in the cells stimulated with HGF (*p* < 0.05, Figure 3(b)).

### 3.5. Zebularine and p38 Specific Inhibitor SB203580 Inhibited Expression of DNMT1

**Figure 4:**
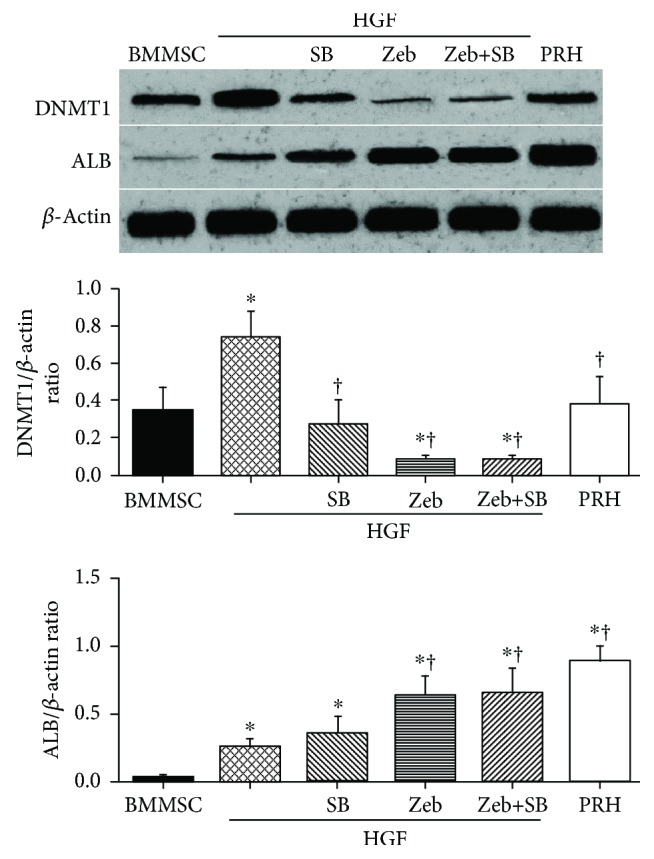
Zebularine and SB203580 inhibited expression of DNMT1 and upregulated expression of ALB in BMMSCs. Noninduced BMMSCs and primary rabbit hepatocytes (PRH) were used as negative or positive controls. HGF increased the expression of DNMT1. HGF-induced expression of DNMT1 was inhibited by zebularine and SB203580. ALB protein expression was regulated by SB203580 and zebularine. The peak of expression of ALB in the induced groups was lower than that in primary rabbit hepatocytes. Asterisk indicates *p* < 0.05 compared with the control group; dagger indicates *p* < 0.05 compared with the HGF group.

Cells were treated with zebularine, SB203580, and zebularine + SB203580 in the presence of HGF. Expression level of DNMT1 protein was also measured via Western blot (Figure 4). HGF increased the expression of DNMT1 (*p* < 0.05). HGF-induced upregulation of DNMT1 was inhibited by zebularine and SB203580 (both *p* < 0.05). Zebularine had a stronger effect on DNMT1 than SB203580 did (*p* < 0.05), though the difference between zebularine and zebularine + SB203580 groups was not significant (*p* > 0.05). DNMT1 level was similar in noninduced BMMSCs and primary rabbit hepatocytes (*p* > 0.05).

**Figure 5:**
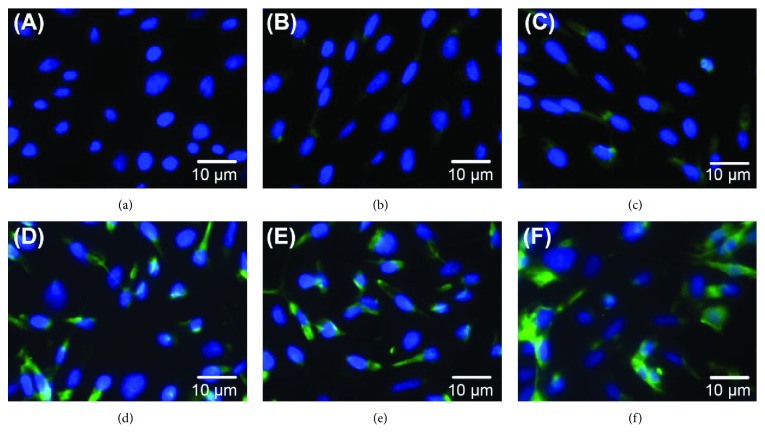
Recruitment of ALB in the cytoplasm of BMMSCs on the 21st day. (a) Noninduced BMMSCs, (b) HGF, (c) HGF + SB203580, (d) HGF + zebularine, (e) HGF + zebularine + SB203580, and (f) primary rabbit hepatocytes. Cells analyzed by immunofluorescence with ALB (green) as a hepatocyte marker and DAPI to stain nuclear (blue). There were (18.67 ± 6.19)%, (26.71 ± 7.31)%, (70.04 ± 6.09)%, (71.82 ± 5.67)%, and (89.36 ± 5.38)% positive cells in HGF, HGF + SB203580, HGF + zebularine, HGF + zebularine + SB203580, and primary rabbit hepatocyte groups, respectively.

We also found that ALB protein expression was upregulated by SB203580 (*p* < 0.05, Figure 4), while the strengthening effect of SB203580 on cell differentiation was weaker than that of zebularine (*p* < 0.05). However, the difference between zebularine and zebularine + SB203580 groups was not significant (*p* < 0.05). Furthermore, the peak of expression of ALB in the induced groups was lower than that in primary rabbit hepatocytes (all *p* < 0.05). Similar results were found on immunofluorescence microscopic evaluation of ALB expression (Figure 5).

### 3.6. ICG Metabolism and Urea Production

**Figure 6:**
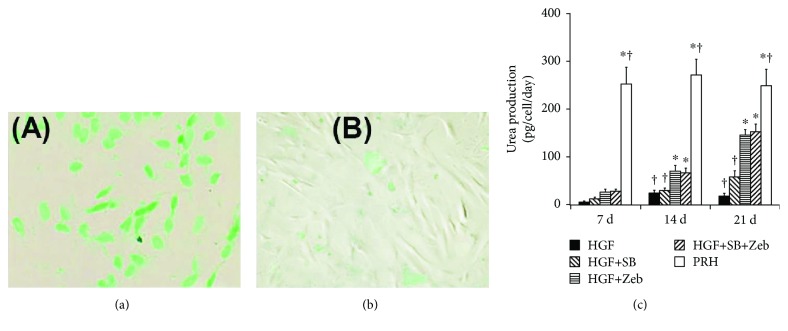
ICG metabolism and urea production. BMMSC-derived cells were positive for ICG after incubation in ICG solution for 30 min (a). ICG in the differentiated cells was released after 6 h (b). Zebularine treatment induced urea production in the cells (c). Asterisk indicates *p* < 0.05 compared with the HGF group; dagger indicates *p* < 0.05 compared with the zebularine group.

Cellular uptake and releasing of ICG, which determines the liver-specific metabolic function, were used to identify differentiated hepatocytes in vitro. Induced cells were positive for ICG after incubation in ICG solution for 30 min (Figure 6(a)). ICG in the differentiated cells was released after 6 h (Figure 6(b)). In addition, urea production was investigated. Zebularine treatment induced urea production in the cells on days 14 and 21 (Figure 6(c), both *p* < 0.05). However, urea production in cells in induced groups was lower than that in primary rabbit hepatocytes (all *p* > 0.05).

## 4. Discussion

Though hepatocyte transplantation is considered to be an alternative to orthotopic liver transplantation, the shortage of donor cells for hepatocyte transplantation has not been resolved. Therefore, the derivation of hepatocytes from stem cells is of value in the creation of an unlimited source of donor cells for hepatocyte transplantation therapy. Nowadays, autologous BMMSCs have been widely applied in the liver repair, but its efficacy is often limited by the low transdifferentiation rate [21–23]. Cytokines, including HGF, epidermal growth factor (EGF), and vascular endothelial growth factor (VEGF) 165 [11], have been shown to induce hepatic differentiation of BMMSCs, though their application is limited by short half-life in vivo and hemodynamic abnormalities and potentially severe side effects when used in large doses.

By activation of methylated genes in stem cells, DNMTi serve as prime candidates for cellular differentiation agents. The effect of zebularine on inhibiting DNA methylation was demonstrated in mammalian stem cell lines [24, 25]. However, a number of studies have investigated the effect of zebularine on hepatic tumors and cardiomyocyte differentiation. It has been reported that zebularine inhibits liver tumor cell proliferation, induces apoptosis, and promotes their maturation and differentiation in a concentration-, time-, and dose-dependent manner [26, 27]. In another study, it has been demonstrated that different doses of zebularine have opposite roles in immunogenicity [28]. In the present study, low concentrations of zebularine could not affect cell viability. When concentration of zebularine increased, the cell viability decreased. This phenomenon may be due to higher doses of zebularine mediating cell cytotoxicity by embedding into DNA and RNA and directly inhibiting cell proliferation. Meanwhile, lower doses of zebularine primarily inhibit DNA methylation, resulting in the recovery of gene normal expression. We could detect a decrease of DNMT1 in BMMSCs by using zebularine. More interestingly, inhibition of DNMT activity by zebularine treatment leads to an increase of AFP and ALB in rabbit cells and therefore support the hepatic differentiation. These data were supported by immunostaining. To the best of our knowledge, this is the first report to show that zebularine leads to remarkable increment in hepatic differentiation of BMMSCs. Also, we observed that zebularine addition induced expression of urea production of and ICG metabolism in our cells, suggesting that zebularine-treated cells possess hepatic-like functionality.

Previous studies have demonstrated that treatment of BMMSCs by zebularine resulted in the expression of cardiac-specific proteins in vitro [29], suggesting that DNA methylation is not the only factor that determines gene expression during cell differentiation process; appropriate microenvironment and the induced culture conditions also have effects on the cell differentiation process. Anyway, combination of zebularine and cell factors is a better method than that of only growth factor supplement for higher transdifferentiation rate, and zebularine could be used as a new method or it can be used in combination with other systems to enhance hepatic differentiation.

The balance between proliferation and differentiation as well as between apoptosis and cell survival is required for DNMTi to promote liver regeneration. Indeed, inhibited HGF signaling, disrupted hepatocyte proliferation, and hepatocyte apoptosis increased the susceptibility of the liver to failure [30]. In hepatic failure and acute liver injury, increased blood HGF level plays an important functional role in liver regeneration but also induces expression of TGF-*β* family [30], which induces apoptosis during fibrogenesis and provides growth control in regeneration processes. Expression of p38 signal was required for TGF-*β*-induced hepatocyte apoptosis. Previous studies have indicated the reciprocal regulation of HGF and p38 MAPK [16, 19, 31]. In this study, the p38 signal was activated in BMMSCs simulated with HGF; the time to peak is 20 min, which is similar to the finding in human BMMSCs [32]. These results suggest that p38 could be involved in the transformation of BMMSCs from different species.

**Figure 7:**
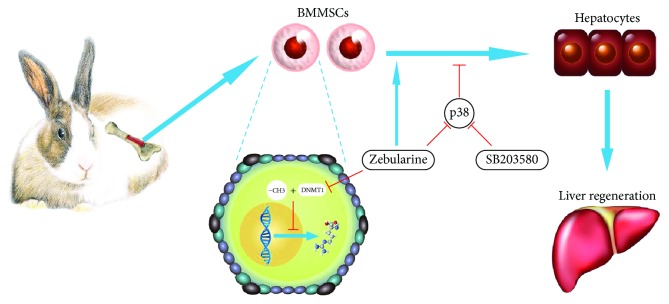
Graphical abstract. The increase in the level of DNMT1 and phosphorylated p38 in BMMSCs was restrained by zebularine. Zebularine leads to remarkable increment in hepatic differentiation of BMMSCs, partly through regulation of HGF-induced p38 activation.

p38 is also involved in regulating of DNMT expression, though its role is often controversial in different cell types and in different laboratories. For example, reports revealed that phosphorylated p38 induces toxic injuries through mediating DNMT1, DNMT3a, and DNMT3b expressions [33, 34], while others documented that activated p38 represses the induction of DNMT1 [35, 36]. In our study, inhibition of p38 by SB203580 significantly decreased the expression of DNMT1. Here, we used SB203580 to determine the specificity of the p38 signaling pathway in mediating hepatic differentiation of BMMSCs. We found that SB203580 inhibited p38 phosphorylation and expression of hepatic-specific proteins induced by HGF, with a weaker force than zebularine. These data demonstrated that the p38 pathway at least partially participates in zebularine-induced hepatic differentiation of rabbit BMMSCs (Figure 7).

There are several questions that remain unanswered. For instance, the maturation degree and substitution function of hepatocyte-like cells from BMMSCs still fell short of primary rabbit hepatocytes. The concentration and induction time needs further improvement in order to obtain better transdifferentiation rate. Besides, we used BMMSCs from rabbit in vitro to explore the potential of zebularine as the differentiation inducer; potential response between zebularine and cells in vivo has not been investigated. Our next research interests may involve the therapeutic potential of hepatocyte-like cells induced by zebularine in vivo in a rabbit model of liver injury, as well as induction of BMMSCs derived from human. Further studies are needed to investigate how zebularine restrains p38 and the detailed interactions between its downstream targets.

In conclusion, we demonstrated that zebularine significantly increases the expression of hepatic-associated marker proteins in BMMSCs and enhances the urea production and ICG metabolic function of these cells. Zebularine exerts its demethylating effect through inhibition of DNNT1, partially by interfering with p38 MAPK signaling. The results obtained from this study would serve as an attempt in the therapeutic march to enhance regeneration of injured liver.

## Data Availability

The data used to support the findings of this study are available from the corresponding author upon request.
